# 2-(2-Fluoro­benzoyl­meth­yl)benzoic acid

**DOI:** 10.1107/S160053680803540X

**Published:** 2008-11-08

**Authors:** Muhammad Tahir Hussain, Tariq Mahmood Babar, Ghulam Qadeer, Nasim Hasan Rama, Ales Ruzicka

**Affiliations:** a Department of Applied Sciences, National Textile University, Faisalabad, Pakistan; b Department of Chemistry, Quaid-i-Azam University, Islamabad 45320, Pakistan; c Department of General and Inorganic Chemistry, Faculty of Chemical Technology, University of Pardubice, Nam. Cs. Legii’ 565, 53210 Pardubice, Czech Republic

## Abstract

In the title compound, C_15_H_11_FO_3_, the aromatic rings are oriented at a dihedral angle of 69.26 (3)°. In the crystal structure, inversion dimers arise from pairs of inter­molecular O—H⋯O hydrogen bonds, and C—H⋯O hydrogen bonds further consolidate the packing. There are also C—H⋯π contacts between the benzoic acid and 2-fluoro­benzene rings.

## Related literature

For the biological activity of isocoumarin and 3,4-dihydro­isocoumarin derivatives, see: Hill (1986 ▶); Napolitano (1997 ▶); Oikawa *et al.* (1997 ▶); Kongsaeree *et al.* (2003 ▶). For bond-length data, see: Allen *et al.* (1987 ▶).
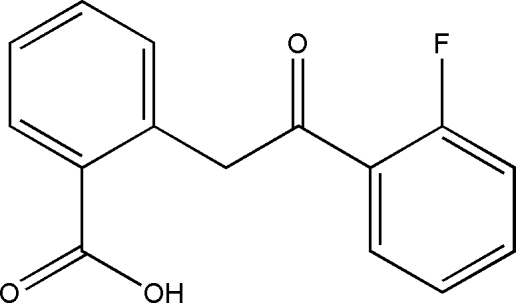


## Experimental

### 

#### Crystal data

C_15_H_11_FO_3_
*M* *_r_* = 258.24
Monoclinic, 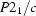

*a* = 8.3011 (6) Å
*b* = 15.3232 (8) Å
*c* = 9.9078 (10) Å
β = 96.942 (8)°
*V* = 1251.02 (17) Å^3^
*Z* = 4
Mo *K*α radiation
μ = 0.10 mm^−1^
*T* = 150 (1) K
0.52 × 0.38 × 0.30 mm

#### Data collection

Bruker–Nonius Kappa CCD area-detector diffractometer
Absorption correction: Gaussian (Coppens, 1970 ▶) *T*_min_ = 0.962, *T*_max_ = 0.975
8420 measured reflections
2782 independent reflections
2117 reflections with *I* > 2σ(*I*)
*R*_int_ = 0.040

#### Refinement

*R*[*F* ^2^ > 2σ(*F* ^2^)] = 0.053
*wR*(*F* ^2^) = 0.136
*S* = 1.12
2782 reflections
172 parameters
H-atom parameters constrained
Δρ_max_ = 0.23 e Å^−3^
Δρ_min_ = −0.29 e Å^−3^

### 

Data collection: *COLLECT* (Hooft, 1998 ▶); cell refinement: *COLLECT* and *DENZO* (Otwinowski & Minor, 1997 ▶); data reduction: *COLLECT* and *DENZO*; program(s) used to solve structure: *SIR92* (Altomare *et al.*, 1994 ▶); program(s) used to refine structure: *SHELXL97* (Sheldrick, 2008 ▶); molecular graphics: *PLATON* (Spek, 2003 ▶); software used to prepare material for publication: *SHELXL97*.

## Supplementary Material

Crystal structure: contains datablocks I, global. DOI: 10.1107/S160053680803540X/hk2566sup1.cif

Structure factors: contains datablocks I. DOI: 10.1107/S160053680803540X/hk2566Isup2.hkl

Additional supplementary materials:  crystallographic information; 3D view; checkCIF report

## Figures and Tables

**Table 1:** Hydrogen-bond geometry (Å, °)

*D*—H⋯*A*	*D*—H	H⋯*A*	*D*⋯*A*	*D*—H⋯*A*
O2—H2⋯O1^i^	0.82	1.80	2.621 (3)	175
C12—H12⋯O3^ii^	0.93	2.46	3.178 (3)	134
C4—H4⋯*Cg*2^iii^	0.93	2.72	3.535 (3)	146
C13—H13⋯*Cg*1^ii^	0.93	3.06	3.868 (3)	146

Symmetry codes: (i) 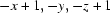; (ii) 
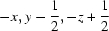
; (iii) 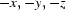. *Cg*1 and *Cg*2 are the centroids of the C2–C7 and C10–C15 rings, respectively.

**Fig. 1.**
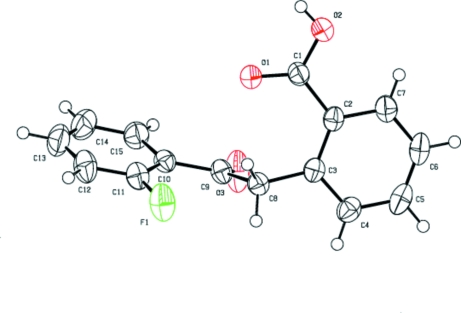
The molecular structure of the title molecule, with the atom-numbering scheme. Displacement ellipsoids are drawn at the 30% probability level.

**Fig. 2.**
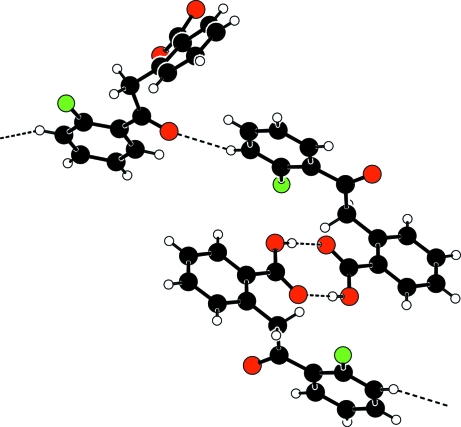
A partial packing diagram. Hydrogen bonds are shown as dashed lines.

**Fig. 3.**
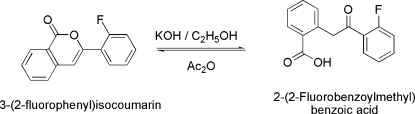
The formation of the title compound.

## supplementary crystallographic information

### Comment

The title compound is an important intermediate in the conversion of isocoumarin to 3,4-dihydroisocoumarin. Derivatives of isocoumarin and 3,4-dihydroisocoumarin display a broad range of biological activity (Hill, 1986; Napolitano, 1997). 3,4-Dihydroisocoumarins are an important class of naturally occurring, biologically active gamma-lactones. Numbers of such dihydroisocoumarins have various uses, ranging from sweetening agents to bactericides, antimalarial, antituberclous, antifungal, antiulcergenic and antitumour (Oikawa *et al.*,  1997; Kongsaeree *et al.*,  2003).

In the title compound (Fig. 1), the bond lengths (Allen *et al.*, 1987) and angles are within normal ranges. Rings A (C2-C7) and B (C10-C15) are, of course, planar and they are oriented at a dihedral angle of 69.26 (3)°. The (O1/O2/C1) moiety is oriented with respect to rings A and B at dihedral angles of 14.54 (4)° and 76.12 (3)°, respectively.

In the crystal structure, intermolecular O-H···O and C-H···O hydrogen bonds (Table 1) link the molecules (Fig. 2), in which they may be effective in the stabilization of the structure. There also exist C—H···π contacts (Table 1) between the benzoic acid and 2-fluorobenzene rings.

### Experimental

3-(2-Fluorophenyl)isocoumarin (6.4 mmol) was dissolved in ethanol (25 ml) and potassium hydroxide (30 ml 5%) was added. The mixture refluxed for about 5 h. After cooling the solvent was evaporated under reduced pressure. Cold water (20 ml) was added and the reaction mixture acidified with hydrochloric acid (5%). The precipitated keto acid was filtered, washed, dried and recrystalized from hot ethanol (yield; 87%, m.p. 404-405 K).

### Refinement

H atoms were positioned geometrically, with O-H = 0.82 Å (for OH) and C-H = 0.93 and 0.97 Å for aromatic and methylene H, respectively, and constrained to ride on their parent atoms with U_iso_(H) = 1.2U_eq_(C,O).

### Crystal data

**Table:**

C_15_H_11_FO_3_	*F*_000_ = 536
*M**_r_* = 258.24	*D*_x_ = 1.371 Mg m^−^^3^
Monoclinic, *P*2_1_/*c*	Melting point: 404(1) K
Hall symbol: -P 2ybc	Mo *K*α radiation λ = 0.71073 Å
*a* = 8.3011 (6) Å	Cell parameters from 8503 reflections
*b* = 15.3232 (8) Å	θ = 1–27.5º
*c* = 9.9078 (10) Å	µ = 0.11 mm^−^^1^
β = 96.942 (8)º	*T* = 150 (1) K
*V* = 1251.02 (17) Å^3^	Block, colorless
*Z* = 4	0.52 × 0.38 × 0.31 mm

### Data collection

**Table:**

Bruker–Nonius Kappa CCD area-detector diffractometer	2782 independent reflections
Radiation source: fine-focus sealed tube	2117 reflections with *I* > 2σ(*I*)
Monochromator: graphite	*R*_int_ = 0.040
Detector resolution: 9.091 pixels mm^-1^	θ_max_ = 27.5º
*T* = 150(1) K	θ_min_ = 2.5º
φ and ω scans	*h* = −9→10
Absorption correction: Gaussian(Coppens, 1970)	*k* = −18→19
*T*_min_ = 0.962, *T*_max_ = 0.975	*l* = −12→12
8420 measured reflections	

### Refinement

**Table:**

Refinement on *F*^2^	Secondary atom site location: difference Fourier map
Least-squares matrix: full	Hydrogen site location: inferred from neighbouring sites
*R*[*F*^2^ > 2σ(*F*^2^)] = 0.053	H-atom parameters constrained
*wR*(*F*^2^) = 0.136	*w* = 1/[σ^2^(*F*_o_^2^) + (0.0382*P*)^2^ + 0.8217*P*] where *P* = (*F*_o_^2^ + 2*F*_c_^2^)/3
*S* = 1.12	(Δ/σ)_max_ < 0.001
2782 reflections	Δρ_max_ = 0.23 e Å^−^^3^
172 parameters	Δρ_min_ = −0.29 e Å^−^^3^
Primary atom site location: structure-invariant direct methods	Extinction correction: none

### Special details

**Table:**

Geometry. All e.s.d.'s (except the e.s.d. in the dihedral angle between two l.s. planes) are estimated using the full covariance matrix. The cell e.s.d.'s are taken into account individually in the estimation of e.s.d.'s in distances, angles and torsion angles; correlations between e.s.d.'s in cell parameters are only used when they are defined by crystal symmetry. An approximate (isotropic) treatment of cell e.s.d.'s is used for estimating e.s.d.'s involving l.s. planes.
Refinement. Refinement of *F*^2^ against ALL reflections. The weighted *R*-factor *wR* and goodness of fit *S* are based on *F*^2^, conventional *R*-factors *R* are based on *F*, with *F* set to zero for negative *F*^2^. The threshold expression of *F*^2^ > σ(*F*^2^) is used only for calculating *R*-factors(gt) *etc*. and is not relevant to the choice of reflections for refinement. *R*-factors based on *F*^2^ are statistically about twice as large as those based on *F*, and *R*- factors based on ALL data will be even larger.

### Fractional atomic coordinates and isotropic or equivalent isotropic displacement parameters (Å^2^)

**Table:**

	*x*	*y*	*z*	*U*_iso_*/*U*_eq_	
F1	0.15542 (18)	−0.18437 (8)	0.10130 (16)	0.0594 (4)	
O1	0.3928 (2)	−0.00007 (12)	0.35571 (16)	0.0573 (5)	
O2	0.61607 (19)	0.07728 (11)	0.40921 (15)	0.0504 (4)	
H2	0.6161	0.0506	0.4809	0.061*	
O3	0.0632 (2)	0.07181 (10)	0.1708 (2)	0.0626 (5)	
C1	0.4909 (2)	0.05329 (13)	0.3244 (2)	0.0361 (4)	
C2	0.4762 (2)	0.09750 (12)	0.1908 (2)	0.0326 (4)	
C3	0.3662 (2)	0.06805 (12)	0.08185 (19)	0.0316 (4)	
C4	0.3530 (3)	0.11624 (15)	−0.0370 (2)	0.0410 (5)	
H4	0.2814	0.0979	−0.1111	0.049*	
C5	0.4429 (3)	0.19136 (15)	−0.0483 (2)	0.0477 (6)	
H5	0.4292	0.2236	−0.1284	0.057*	
C6	0.5523 (3)	0.21865 (14)	0.0580 (2)	0.0468 (5)	
H6	0.6140	0.2686	0.0499	0.056*	
C7	0.5704 (3)	0.17144 (13)	0.1770 (2)	0.0397 (5)	
H7	0.6464	0.1889	0.2485	0.048*	
C8	0.2660 (2)	−0.01340 (13)	0.0886 (2)	0.0335 (4)	
H8A	0.3338	−0.0593	0.1325	0.040*	
H8B	0.2282	−0.0325	−0.0032	0.040*	
C9	0.1223 (2)	0.00021 (12)	0.1651 (2)	0.0344 (4)	
C10	0.0483 (2)	−0.07466 (12)	0.2322 (2)	0.0330 (4)	
C11	0.0665 (2)	−0.16193 (13)	0.2005 (2)	0.0395 (5)	
C12	−0.0064 (3)	−0.22847 (15)	0.2627 (3)	0.0520 (6)	
H12	0.0071	−0.2862	0.2371	0.062*	
C13	−0.1001 (3)	−0.20839 (18)	0.3633 (3)	0.0599 (7)	
H13	−0.1490	−0.2527	0.4079	0.072*	
C14	−0.1213 (3)	−0.12269 (19)	0.3988 (3)	0.0603 (7)	
H14	−0.1854	−0.1092	0.4667	0.072*	
C15	−0.0491 (3)	−0.05654 (16)	0.3331 (2)	0.0469 (5)	
H15	−0.0654	0.0011	0.3573	0.056*	

### Atomic displacement parameters (Å^2^)

**Table:**

	*U*^11^	*U*^22^	*U*^33^	*U*^12^	*U*^13^	*U*^23^
F1	0.0607 (9)	0.0356 (7)	0.0886 (11)	−0.0018 (6)	0.0356 (8)	−0.0117 (7)
O1	0.0577 (10)	0.0698 (12)	0.0414 (9)	−0.0320 (9)	−0.0064 (7)	0.0151 (8)
O2	0.0525 (9)	0.0544 (10)	0.0422 (8)	−0.0221 (8)	−0.0034 (7)	0.0060 (7)
O3	0.0532 (10)	0.0293 (8)	0.1121 (15)	0.0072 (7)	0.0381 (10)	0.0056 (9)
C1	0.0367 (10)	0.0344 (10)	0.0375 (10)	−0.0062 (8)	0.0057 (8)	−0.0028 (8)
C2	0.0350 (10)	0.0281 (9)	0.0369 (10)	0.0002 (7)	0.0126 (8)	−0.0010 (7)
C3	0.0277 (9)	0.0324 (10)	0.0365 (10)	0.0044 (7)	0.0108 (8)	0.0008 (8)
C4	0.0351 (10)	0.0487 (12)	0.0402 (11)	0.0050 (9)	0.0086 (8)	0.0075 (9)
C5	0.0467 (12)	0.0463 (12)	0.0538 (13)	0.0066 (10)	0.0210 (11)	0.0179 (10)
C6	0.0495 (13)	0.0339 (11)	0.0614 (14)	−0.0047 (9)	0.0245 (11)	0.0058 (10)
C7	0.0410 (11)	0.0350 (11)	0.0455 (12)	−0.0050 (8)	0.0146 (9)	−0.0054 (9)
C8	0.0329 (10)	0.0320 (10)	0.0360 (10)	−0.0013 (8)	0.0050 (8)	−0.0019 (8)
C9	0.0309 (9)	0.0291 (10)	0.0432 (11)	0.0000 (8)	0.0046 (8)	−0.0005 (8)
C10	0.0293 (9)	0.0331 (10)	0.0363 (10)	−0.0006 (7)	0.0023 (7)	0.0001 (8)
C11	0.0325 (10)	0.0343 (10)	0.0522 (13)	0.0020 (8)	0.0068 (9)	0.0013 (9)
C12	0.0460 (12)	0.0328 (11)	0.0772 (17)	−0.0012 (9)	0.0074 (12)	0.0106 (11)
C13	0.0589 (15)	0.0561 (15)	0.0653 (16)	−0.0107 (12)	0.0103 (13)	0.0231 (13)
C14	0.0649 (17)	0.0703 (18)	0.0504 (14)	−0.0106 (13)	0.0257 (12)	0.0032 (12)
C15	0.0514 (13)	0.0455 (12)	0.0459 (12)	−0.0052 (10)	0.0142 (10)	−0.0059 (10)

### Geometric parameters (Å, °)

**Table:**

F1—C11	1.343 (2)	C8—H8A	0.9700
O1—C1	1.220 (2)	C8—H8B	0.9700
O2—C1	1.308 (2)	C9—O3	1.206 (2)
O2—H2	0.8199	C9—C8	1.503 (3)
C2—C1	1.479 (3)	C9—C10	1.495 (3)
C2—C3	1.402 (3)	C10—C11	1.386 (3)
C2—C7	1.393 (3)	C10—C15	1.388 (3)
C3—C4	1.383 (3)	C11—C12	1.370 (3)
C3—C8	1.506 (3)	C12—C13	1.371 (4)
C4—C5	1.384 (3)	C12—H12	0.9299
C4—H4	0.9300	C13—H13	0.9300
C5—C6	1.370 (3)	C14—C13	1.376 (4)
C5—H5	0.9300	C14—H14	0.9300
C6—H6	0.9299	C15—C14	1.380 (3)
C7—C6	1.376 (3)	C15—H15	0.9300
C7—H7	0.9300		
			
C1—O2—H2	109.5	C9—C8—H8B	109.1
O1—C1—O2	121.87 (19)	C3—C8—H8B	109.1
O1—C1—C2	123.30 (18)	H8A—C8—H8B	107.8
O2—C1—C2	114.80 (17)	O3—C9—C10	119.08 (18)
C7—C2—C3	120.47 (18)	O3—C9—C8	120.14 (18)
C7—C2—C1	118.31 (18)	C10—C9—C8	120.77 (16)
C3—C2—C1	121.18 (17)	C11—C10—C15	116.41 (19)
C4—C3—C2	117.49 (18)	C11—C10—C9	125.32 (18)
C4—C3—C8	119.50 (18)	C15—C10—C9	118.26 (18)
C2—C3—C8	123.00 (17)	F1—C11—C12	116.77 (19)
C3—C4—C5	121.6 (2)	F1—C11—C10	119.81 (18)
C3—C4—H4	119.2	C12—C11—C10	123.4 (2)
C5—C4—H4	119.2	C11—C12—C13	118.7 (2)
C6—C5—C4	120.4 (2)	C11—C12—H12	120.7
C6—C5—H5	119.7	C13—C12—H12	120.6
C4—C5—H5	119.9	C12—C13—C14	120.1 (2)
C5—C6—C7	119.4 (2)	C12—C13—H13	120.0
C5—C6—H6	120.4	C14—C13—H13	119.9
C7—C6—H6	120.2	C13—C14—C15	120.2 (2)
C6—C7—C2	120.5 (2)	C13—C14—H14	120.0
C6—C7—H7	119.7	C15—C14—H14	119.8
C2—C7—H7	119.8	C14—C15—C10	121.1 (2)
C9—C8—C3	112.56 (16)	C14—C15—H15	119.5
C9—C8—H8A	109.1	C10—C15—H15	119.4
C3—C8—H8A	109.1		

### Hydrogen-bond geometry (Å, °)

**Table:**

*D*—H···*A*	*D*—H	H···*A*	*D*···*A*	*D*—H···*A*
O2—H2···O1^i^	0.82	1.80	2.621 (3)	175
C12—H12···O3^ii^	0.93	2.46	3.178 (3)	134
C4—H4···Cg2^iii^	0.93	2.72	3.535 (3)	146
C13—H13···Cg1^ii^	0.93	3.06	3.868 (3)	146

Symmetry codes: (i) −*x*+1, −*y*, −*z*+1; (ii) −*x*, *y*−1/2, −*z*+1/2; (iii) −*x*, −*y*, −*z*.
